# Sex and *APOE* ε4 allele differences in longitudinal white matter microstructure in multiple cohorts of aging and Alzheimer's disease

**DOI:** 10.1002/alz.14343

**Published:** 2024-12-22

**Authors:** Amalia Peterson, Aditi Sathe, Dimitrios Zaras, Yisu Yang, Alaina Durant, Kacie D. Deters, Niranjana Shashikumar, Kimberly R. Pechman, Michael E. Kim, Chenyu Gao, Nazirah Mohd Khairi, Zhiyuan Li, Tianyuan Yao, Yuankai Huo, Logan Dumitrescu, Katherine A. Gifford, Jo Ellen Wilson, Francis E. Cambronero, Shannon L. Risacher, Lori L. Beason‐Held, Yang An, Konstantinos Arfanakis, Guray Erus, Christos Davatzikos, Duygu Tosun, Arthur W. Toga, Paul M. Thompson, Elizabeth C. Mormino, Mohamad Habes, Di Wang, Panpan Zhang, Kurt Schilling, Alzheimer's Disease Neuroimaging Initiative (ADNI), The BIOCARD Study Team, The Alzheimer's Disease Sequencing Project (ADSP), Marilyn Albert, Walter Kukull, Sarah A. Biber, Bennett A. Landman, Sterling C. Johnson, Julie Schneider, Lisa L. Barnes, David A. Bennett, Angela L. Jefferson, Susan M. Resnick, Andrew J. Saykin, Timothy J. Hohman, Derek B. Archer

**Affiliations:** ^1^ Vanderbilt Memory and Alzheimer's Center Vanderbilt University School of Medicine Nashville Tennessee USA; ^2^ Department of Neurology Vanderbilt University Medical Center Nashville Tennessee USA; ^3^ Department of Integrative Biology and Physiology University of California Los Angeles California USA; ^4^ Department of Computer Science Vanderbilt University Nashville Tennessee USA; ^5^ Department of Electrical and Computer Engineering Vanderbilt University Nashville Tennessee USA; ^6^ Vanderbilt Genetics Institute Vanderbilt University Medical Center Nashville Tennessee USA; ^7^ Vanderbilt Brain Institute Vanderbilt University Medical Center Nashville Tennessee USA; ^8^ Department of Psychiatry and Behavioral Sciences Center for Cognitive Medicine, Vanderbilt University Medical Center Nashville Tennessee USA; ^9^ Veteran's Affairs, Geriatric Research, Education and Clinical Center Tennessee Valley Healthcare System Nashville Tennessee USA; ^10^ Department of Radiology and Imaging Sciences Indiana University School of Medicine Indianapolis Indiana USA; ^11^ Indiana Alzheimer's Disease Research Center Indiana University School of Medicine Indianapolis Indiana USA; ^12^ Laboratory for Behavioral Neuroscience National Institute on Aging, National Institutes of Health Baltimore Maryland USA; ^13^ Department of Biomedical Engineering Illinois Institute of Technology Chicago Illinois USA; ^14^ Rush Alzheimer's Disease Center Rush University Medical Center Chicago Illinois USA; ^15^ Department of Diagnostic Radiology Rush University Medical Center Chicago Illinois USA; ^16^ Department of Radiology University of Pennsylvania Philadelphia Pennsylvania USA; ^17^ Department of Radiology and Biomedical Imaging University of California San Francisco San Francisco California USA; ^18^ Laboratory of Neuroimaging, USC Stevens Institute of Neuroimaging and Informatics Keck School of Medicine, University of Southern California Los Angeles California USA; ^19^ Imaging Genetics Center, Mark and Mary Stevens Institute for Neuroimaging and Informatics Keck School of Medicine, University of Southern California Marina del Rey California USA; ^20^ Department of Neurology and Neurological Sciences Stanford University School of Medicine Stanford California USA; ^21^ Neuroimage Analytics Laboratory and Biggs Institute Neuroimaging Core, Glenn Biggs Institute for Neurodegenerative Disorders University of Texas Health Science Center at San Antonio San Antonio Texas USA; ^22^ Department of Biostatistics Vanderbilt University Medical Center Nashville Tennessee USA; ^23^ Department of Radiology & Radiological Sciences Vanderbilt University Medical Center Nashville Tennessee USA; ^24^ Vanderbilt University Institute of Imaging Science Vanderbilt University Medical Center Nashville Tennessee USA; ^25^ Department of Neurology Johns Hopkins School of Medicine Baltimore Maryland USA; ^26^ National Alzheimer's Coordinating Center University of Washington Seattle Washington USA; ^27^ Department of Biomedical Engineering Vanderbilt University Nashville Tennessee USA; ^28^ Wisconsin Alzheimer's Disease Research Center University of Wisconsin School of Medicine and Public Health Madison Wisconsin USA; ^29^ Wisconsin Alzheimer's Institute University of Wisconsin School of Medicine and Public Health Madison Wisconsin USA

**Keywords:** aging, Alzheimer's disease, sex differences, white matter disease

## Abstract

**INTRODUCTION:**

The effects of sex and apolipoprotein E (*APOE*)—Alzheimer's disease (AD) risk factors—on white matter microstructure are not well characterized.

**METHODS:**

Diffusion magnetic resonance imaging data from nine well‐established longitudinal cohorts of aging were free water (FW)–corrected and harmonized. This dataset included 4741 participants (age = 73.06 ± 9.75) with 9671 imaging sessions over time. FW and FW‐corrected fractional anisotropy (FA_FWcorr_) were used to assess differences in white matter microstructure by sex and *APOE* ε4 carrier status.

**RESULTS:**

Sex differences in FA_FWcorr_ in projection tracts and *APOE* ε4 differences in FW limbic and occipital transcallosal tracts were most pronounced.

**DISCUSSION:**

There are prominent differences in white matter microstructure by sex and *APOE* ε4 carrier status. This work adds to our understanding of disparities in AD. Additional work to understand the etiology of these differences is warranted.

**Highlights:**

- Sex and apolipoprotein E (*APOE*) ε4 carrier status relate to white matter microstructural integrity.
- Females generally have lower free water–corrected fractional anisotropy compared to males.
- *APOE* ε4 carriers tended to have higher free water than non‐carriers.

## 1 BACKGROUND

Although Alzheimer's disease (AD) is traditionally associated with gray matter pathology, emerging data highlights distinct white matter abnormalities in AD, including axonal loss,^1^ demyelination,^2^ and microglial activation,^3^ that can occur up to 20 years before symptom onset.^4^ In familial AD, in which amyloid and tau accumulate at a young age and there are few co‐occurring pathologies or vascular risk factors,^5^ white matter microstructural damage precedes detectable changes in both hippocampal volume and clinical symptoms.^6^ This suggests that white matter damage in AD is not solely due to microvascular disease, and at least in part relates to the underlying pathological mechanisms driving the development of AD. Females, self‐identified non‐Hispanic Black adults, and apolipoprotein E (*APOE*) ε4 carriers are at greater risk for clinical AD, but the pathways by which this occurs are still being elucidated. Examining how white matter microstructure differs in these populations is important for understanding disparities in AD and developing targeted interventions.

Two thirds of people living with AD are women.^7^ There are striking sex differences in AD risk factors, clinical presentation, and neuropathological burden, though the underlying reasons for these differences are not well understood.^8^ Several studies,^9^, ^10^, ^11^ but not all,^12^, ^13^, ^14^ have found sex differences in white matter microstructure. However, many of these studies are limited by small sample size,^12^, ^13^, ^14^ cross‐sectional design,^12^, ^13^ and use of conventional diffusion tensor imaging (DTI),^9^, ^14^ thus limiting our understanding of how the trajectory of white matter microstructure in aging differs by sex.

*APOE* ε4 is the strongest genetic risk factor for late‐onset AD.^15^ The association between *APOE* ε4 and AD risk is stronger in females and non‐Hispanic White adults compared to males^16^ and non‐Hispanic Black adults.^17^ *APOE* has an important role in cholesterol transport and lipid metabolism and may play a role in myelin maintenance,^18^ but the effect of *APOE* ε4 on white matter microstructure is not well established.^19^, ^20^

Racial categories are social constructs that serve as proxies for sociocultural forces, including social, economic, and environmental factors, that ultimately affect cognition.^21^ Non‐Hispanic Black Americans are twice as likely to have AD and related dementias compared to non‐Hispanic White Americans,^7^ but are less likely to have amyloid pathology.^22^, ^23^ This suggests that non‐amyloid pathways impacted by social and cultural factors also contribute to cognitive impairment. Whether sex and *APOE* ε4 carrier status differentially affect white matter microstructure in Black and White adults is unknown.

To date, most studies of white matter microstructural abnormalities use conventional DTI. Conventional DTI is derived from a single tensor model and is confounded by partial volume effects, such that each voxel contains both tissue and fluid compartments. Free water (FW) elimination is a two‐tensor DTI model that allows for the separation of fluid (FW) and tissue (FW‐corrected) components, correcting for partial volume,^24^ to give an FW‐corrected fractional anisotropy (FA_FWcorr_) metric. Importantly, FW metrics are considered more sensitive to abnormal brain aging.^25^, ^26^, ^27^ While previous studies have found lower FA_FWcorr_ and higher FW in limbic, association, and transcallosal (TC) tracts^25^, ^26^, ^28^, ^29^, ^30^ associated with cognitive impairment and AD, there has yet to be a large‐scale analysis leveraging FW measures to understand how sex and *APOE* ε4 carrier status are associated with longitudinal white matter microstructure.

The goal of this study is to provide the most comprehensive picture to date of the effects of sex and *APOE* ε4 carrier status on white matter microstructure throughout aging and AD using nine well‐characterized cohorts of older adults. In 4741 participants, leveraging 9671 harmonized longitudinal imaging sessions, we hypothesized that females and *APOE* ε4–carrying participants would have lower FA_FWcorr_ and higher FW and that these differences would be pronounced in limbic, association, and TC tracts.

## 2 METHODS

### 2.1 Participants

The present study used data from participants in the Alzheimer's Disease Neuroimaging Initiative (ADNI), the Baltimore Longitudinal Study of Aging (BLSA), the Biomarkers of Cognitive Decline Among Normal Individuals (BIOCARD) study, the National Alzheimer's Coordinating Center (NACC) data set, the Religious Orders Study/Rush Memory and Aging Project/Minority Aging Research Study (ROS/MAP/MARS), Vanderbilt Memory and Aging Project (VMAP), and the Wisconsin Registry of Alzheimer's Prevention (WRAP) cohorts.

ADNI (www.adni.loni.usc.edu), begun in 2003, was designed to assess brain structure and function using serial magnetic resonance imaging (MRI), other biological markers, and clinical and neuropsychological assessments.^31^ Three ADNI phases (ADNI‐GO, ADNI 2, and ADNI 3) were included in the present study. The BLSA was designed to assess physical and cognitive measures in a community‐dwelling cohort.^32^ Behavioral assessments began in 1994 and included dementia‐free participants aged 55 to 85. From 2006 to 2018, BLSA MRI data were collected on a 1.5T scanner and, beginning in 2009, MRIs were performed with a single 3T MRI scanner. Data from the BLSA cohort are available upon request by a proposal submission through the BLSA website (www.blsa.nih.gov). BIOCARD, begun in 1995, includes participants who were middle aged and cognitively intact at baseline. The study stopped in 2005 and was reestablished in 2009 with annual assessments.^33^ NACC maintains a database of participant information collected from past and present National Institute on Aging‐funded Alzheimer's Disease Research Centers.^34^ ROS/MAP/MARS are longitudinal, epidemiologic clinical–pathologic cohort studies that were designed to characterize common chronic conditions of aging and the neuropathological basis of cognitive impairment. The ROS was started in 1994 and enrolled older Catholic priests, nuns, and brothers from states across the United States.^35^ The MAP started in 1997 and enrolled older men and women in the Chicagoland area.^35^ The MARS was started in 2004 and enrolled older adults who self‐identify as Black in the Chicagoland area.^36^ Notably, the three cohorts are managed by a single team with a large common core of data at the item level with imaging managed through a single pipeline allowing efficient merging of data.^37^ VMAP began in 2012 with the goal of understanding the relationship between vascular and brain health.^38^ WRAP, begun in 2001, is a study of midlife adults enriched with persons with a parental history of AD.^39^ For each cohort, demographic and clinical covariates were required for inclusion, including age, sex, educational attainment, race/ethnicity, *APOE* haplotype status (ε2, ε3, ε4), and cognitive diagnosis (cognitively unimpaired, mild cognitive impairment, AD). Each cohort had its own inclusion/exclusion criteria. Participants were included in this study if they had diffusion MRI (dMRI) data, demographic and clinical data, were 50+ years old, identified as non‐Hispanic White or Black, and passed neuroimaging quality control procedures. Across all cohorts, written informed consent was provided by participants, and research was conducted in accordance with approved institutional review board (IRB) protocols. Secondary analysis of these data was approved by the Vanderbilt University IRB. Table 1 provides an overview of the ADNI, BIOCARD, BLSA, NACC, ROS/MAP/MARS, VMAP and WRAP sample sizes, demographic information, and health characteristics.

**RESEARCH IN CONTEXT**

1. **Systematic review**: The authors used PubMed and Google Scholar to review literature that used conventional and free water (FW)–corrected microstructural metrics to evaluate sex and apolipoprotein E (*APOE*) ε4 differences in white matter microstructure. No large‐scale FW‐corrected analysis has been performed.
2. **Interpretation**: Sex was associated most strongly with FW‐corrected fractional anisotropy while *APOE* ε4 status was associated with FW metrics. Association, projection, limbic, and occipital transcallosal tracts showed the greatest differences.
3. **Future directions**: Future studies to determine the biological and social pathways that lead to sex and *APOE* ε4 differences are warranted.

**TABLE 1 alz14343-tbl-0001:** Demographic and health characteristics by cohort.

	Cohort						
Measure	ADNI	BIOCARD	BLSA	NACC	ROS/MAP/MARS	VMAP	WRAP
***Cohort Characteristics***							
Total number of participants	956	168	728	964	1117	514	294
Total number of sessions	2109	364	1870	1211	2486	1201	430
Average number of visits	2.21 (1.48)	2.17 (0.96)	2.70 (1.90)	1.26 (0.55)	2.23 (1.40)	2.34 (1.36)	1.48 (0.71)
Longitudinal follow‐up (years)	2.66 (1.94)	2.84 (1.09)	3.97 (2.17)	1.60 (0.87)	4.31 (2.52)	2.99 (1.40)	3.46 (1.76)
***Demographic characteristics***							
Age at baseline (years)	73.49 (7.77)	71.73 (7.44)	71.15 (10.23)	71.82 (10.41)	79.55 (7.27)	70.05 (8.85)	61.92 (6.22)
Sex (% female)	50.63	62.50	54.53	58.92	77.26	49.61	65.99
Education (years)	16.28 (2.53)	17.38 (2.25)	16.94 (2.38)	15.32 (3.11)	15.88 (3.22)	16.06 (2.48)	16.67 (2.83)
Race (% non‐Hispanic White)	88.28	98.81	72.80	85.58	76.28	88.33	98.64
*APOE* ε4 (% carriers)	41.29	32.73	28.36	44.52	24.85	36.24	34.90
*APOE* ε2 (% carriers)	9.76	13.33	17.51	14.38	15.64	15.25	13.73
*APOE* ε2/ε4 (% carriers)	1.85	0.61	2.60	3.51	2.80	3.37	3.14
Cognitive status at baseline (% cognitively unimpaired)	48.33	79.76	98.76	61.83	80.75	75.10	98.64
Longitudinal cognitive status converters (*n*)	526	41	45	378	316	157	4

*Note*: Values denoted as mean (standard deviation) or frequency.

Abbreviations: ADNI, Alzheimer's Disease Neuroimaging Initiative; *APOE*, apolipoprotein E; BLSA, Baltimore Longitudinal Study of Aging; BIOCARD, Biomarkers of Cognitive Decline Among Normal Individuals; NACC, National Alzheimer's Coordinating Center; ROS, Religious Orders Study; MAP, Rush Memory and Aging Project; MARS, Minority Aging Research Study; VMAP, Vanderbilt Memory & Aging Project; WRAP, Wisconsin Registry for Alzheimer's Prevention.

### 2.2 Diffusion MRI acquisition and preprocessing

All longitudinal dMRI data were preprocessed using the *PreQual* automated pipeline for denoising, slice‐wise outlier imputation, data quality assurance, and to correct for motion, eddy current, and susceptibility‐induced distortions.^40^, ^41^ After *PreQual* preprocessing, the cleaned data was input into DTIFIT. Simultaneously, the cleaned data was input into MATLAB code^24^ to calculate FW and FA_FWcorr_. After map generation, we created a standard space representation by non‐linearly registering (i.e., symmetric normalization and linear interpolation in ANTs)^42^ the conventional FA (FA_CONV_) map to the FMRIB58_FA atlas. The resulting warp was applied to the FW and FA_FWcorr_ maps, which were used in all subsequent analyses.[Table alz14343-tbl-0001]

### 2.3 White matter tractography templates

**FIGURE 1:**
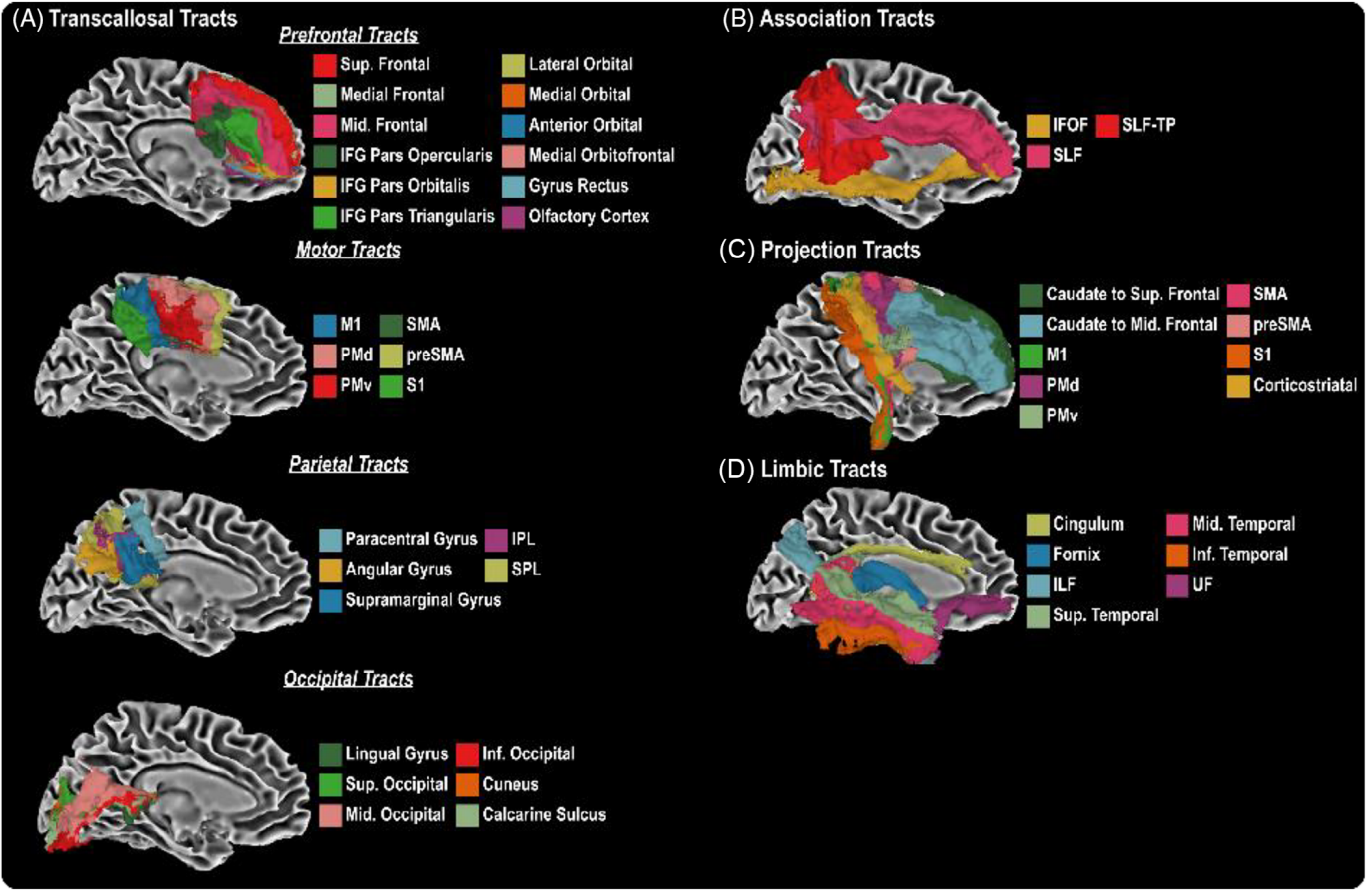
Forty‐eight white matter tractography templates were used in the present study, and can be grouped into TC (A), association (B), projection (C), and limbic tracts (D). IFG, inferior frontal gyrus; IFOF, inferior fronto‐occipital fasciculus; ILF, inferior longitudinal fasciculus; IPL, inferior parietal lobe; M1, primary motor cortex; PMd, dorsal premotor; PMv, ventral premotor; S1, primary somatosensory cortex; SLF, superior longitudinal fasciculus; SLF‐TP, temporoparietal superior longitudinal fasciculus; SMA, supplementary motor area; SPL, superior parietal lobe; TC, transcallosal; UF, uncinate fasciculus.

Consistent with prior work,^25^, ^43^, ^44^, ^45^, ^46^ we used freely accessible tractography templates (https://github.com/VUMC‐VMAC/Tractography_Templates) to assess the white matter microstructure in our cohort (Figure 1). In total, we used 48 tract templates from seven different tract types, including association, limbic, projection, motor TC, occipital TC, parietal TC, and prefrontal TC areas. For each cohort, age‐regressed outlier removal was conducted in which imaging sessions were removed if at least five datapoints were five standard deviations from the mean. Datapoints are collated from the FW, and FW‐corrected axial diffusivity (AxD_FWcorr_), radial diffusivity (RD_FWcorr_), mean diffusivity (MD_FWcorr_), and FA_FWcorr_ metrics across all 48 white matter tracts.

### 2.4 Diffusion MRI data harmonization

A region of interest–based approach was used to quantify mean FW and FA_FWcorr_ (and all other possible) microstructural metrics across all tractography templates for each participant, resulting in a total of 432 (9 microstructural metrics × 48 tracts) unique values for each imaging session. These values were harmonized using the *Longitudinal ComBat* technique in R, controlling for protocol‐specific batch combinations (see Table S1 in supporting information for all 35 batches).^47^ A conservative harmonization approach was used to control for between‐cohort effects, including mean‐centered age, mean‐centered age squared, sex, and diagnosis at baseline. We also included interactions of mean‐centered age and converter status (i.e., cognitively unimpaired, cognitively impaired at some point) and mean‐centered age squared and converter status. The harmonized values were then mean centered and used in all subsequent statistical analyses. Harmonized, tract‐wise means and standard deviations are provided in Table S2 in supporting information for each possible stratification (all participants, men, women, *APOE* ε4 carrier, *APOE* ε4 non‐carrier, non‐Hispanic White, non‐Hispanic Black) and all conventional and FW‐corrected DTI metrics for each individual cohort.

### 2.5 Statistical analyses

All statistical analyses were performed in R (version 4.1.0), and age was mean centered before analysis. Covariates included age at baseline in addition to sex, race/ethnicity, and *APOE* ε4 carrier status. To assess covariate differences in sex, race/ethnicity, and *APOE* ε4, we first evaluated normality using the Shapiro–Wilk test. For normally distributed continuous variables, we tested for homogeneity of variances using the Levene test and performed analysis of variance (ANOVA) when variances were equal; otherwise, a Welch ANOVA was used. For non‐normal distributions, the Kruskal–Wallis test was applied. For categorical variables, we used the chi‐square test, opting for the Fisher exact test when expected frequencies were low. Linear regression models (*stats* R package) were used to determine whether sex or *APOE* ε4 carrier status was associated with cross‐sectional FA_FWcorr_ and FW. Linear mixed‐effects regression models (*nlme* R package) were used to determine whether sex or *APOE* ε4 carrier status was associated with longitudinal FA_FWcorr_ and FW. We controlled for the random aging effects for each participant (i.e., ∼ 1 + age|participant). Models were repeated with a *sex *×* APOE* ε4 interaction term. The reference level for sex was males, whereas the reference level for *APOE* ε4 was non‐carrier status. In our main analysis, multiple corrections were made across microstructural metrics (FA_FWcorr_ and FW) and all white matter tracts. For each main effect and interaction analysis, there were 96 comparisons. Statistics were also aggregated by tract type to determine whether basic tract function was associated with the main effects of sex and *APOE* ε4 carrier status.

Sensitivity analyses were conducted after including a diagnosis covariate, and additional sensitivity analyses were conducted using all possible conventional DTI metrics (FA_CONV_, axial diffusivity [AxD_CONV_], radial diffusivity [RD_CONV_], mean diffusivity [MD_CONV_]) and FW‐corrected DTI metrics (AxD_FWcorr_, RD_FWcorr_, MD_FWcorr_). For the cross‐sectional analysis, race‐stratified sensitivity analyses were conducted, but there was not sufficient longitudinal non‐Hispanic Black data for longitudinal sensitivity analyses. Significance was set a priori as *α* = 0.05 and corrected for multiple comparisons using the false discovery rate method.

## 3 RESULTS

### 3.1 Demographic differences

Females in the study had lower levels of education (χ^2 ^= 115.18, *P* < 2.2 × 10^−16^), were more likely to be cognitively unimpaired at baseline (χ^2 ^= 91.92, *P* < 2.2 × 10^−16^), and were more likely to be longitudinal diagnosis converters (χ^2 ^= 81.30, *P* < 2.2 × 10^−16^) compared to men. *APOE* ε4 carriers were younger at study entry (χ^2 ^= 59.97, *P* = 4.43 × 10^−14^), were more likely to have cognitive impairment at baseline (χ^2 ^= 138.32, *P* < 2.2 × 10^−16^), and were more likely to be longitudinal diagnosis converters (χ^2 ^= 92.34, *P* < 2.2 × 10^−16^) compared to *APOE* ε4 non‐carriers. Non‐Hispanic Black participants were younger at study entry (χ^2 ^= 11.61, *P* = 6.55 × 10^−4^), had lower levels of education (χ^2 ^= 44.05, *P* = 3.21 × 10^−11^), were more likely to be cognitively unimpaired at study entry (χ^2 ^= 19.74, *P* = 5.18 × 10^−5^), and were less likely to be longitudinal diagnosis converters (χ^2 ^= 20.83, *P* = 5.03 × 10^−6^) compared to non‐Hispanic White participants.

### 3.2 Sex associations with FA_FWcorr_ and FW

**FIGURE 2:**
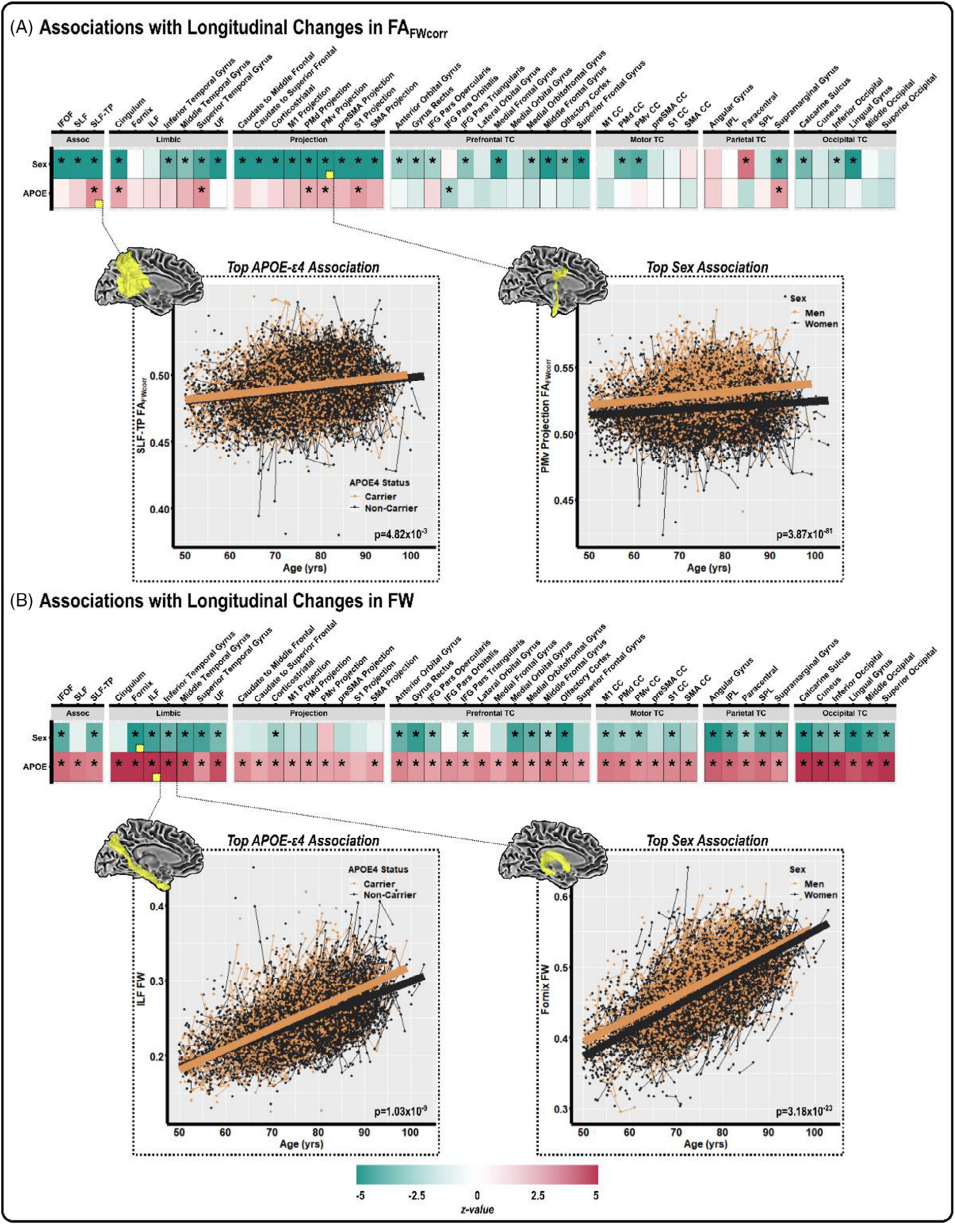
The main effects of sex and *APOE* ε4 on change in white matter microstructure. Linear mixed effects regression was conducted to determine the association of sex and *APOE* ε4 positivity on change in FA_FWcorr_ (A) and change in FW (B). For both measures, heatmaps are grouped by tract type and illustrate the test statistic (i.e., *z* value) for each independent regression analysis. The top sex and *APOE* ε4 associations for both measures are illustrated with spaghetti plots. *Tracts surviving correction for multiple comparisons. *APOE*, apolipoprotein E; CC, corpus callosum; FA_FWcorr_, free water‐corrected fractional anisotropy; FW, free water; IFG, inferior frontal gyrus; IFOF, inferior fronto‐occipital fasciculus; ILF, inferior longitudinal fasciculus; IPL, inferior parietal lobe; M1, primary motor cortex; PMd, dorsal premotor; PMv, ventral premotor; S1, primary somatosensory cortex; SLF, superior longitudinal fasciculus; SLF‐TP, temporoparietal superior longitudinal fasciculus; SMA, supplementary motor area; SPL, superior parietal lobe; TC, transcallosal; UF, uncinate fasciculus.

All results for sex‐specific cross‐sectional and longitudinal models can be found in Table S3 in supporting information. As illustrated in Figure 2A, females had lower FA_FWcorr_ over time relative to males across many white matter tracts. Table 2 summarizes the top five associations for sex, emphasizing the prominence of projection white matter tracts. Notably, the strongest association was observed in the ventral premotor projection tract, as depicted in the spaghetti plot within Figure 2A (*P* = 3.87 × 10^−81^). Figure 2B illustrates the main effects of sex on FW, highlighting significant associations across many limbic, parietal TC, and occipital TC white matter tracts. In all white matter tracts that showed a difference in FW by sex, females had lower FW longitudinally relative to males. The strongest association was observed in the fornix, as depicted in Figure 2B (*P* = 3.18 × 10^−23^). Cross‐sectional results were similar to those seen in longitudinal analyses. In cross‐sectional race‐stratified analyses, there were sex differences in non‐Hispanic Black adults in seven tracts, primarily FA_FWcorr_ in projection tracts, such that females had a lower FA_FWcorr_ over time (Table S4 in supporting information). There were more global differences in non‐Hispanic White adults by sex in both FA_FWcorr_ and FW metrics, but this is likely because we were more powered in the non‐Hispanic White analysis.

**TABLE 2:** Top sex and *APOE* ε4 main effects on longitudinal white matter microstructure.

Tract	Tract type	Outcome	*β* (SE)	*z* value	*p* value
***Main effects of sex***					
PMv projection	Projection	FA_FWcorr_	−9.82 × 10^−3^ (5.08 × 10^−4^)	−19.31	3.87 × 10^−81^
S1 projection	Projection	FA_FWcorr_	−9.13 × 10^−3^ (6.86 × 10^−4^)	−13.31	9.46 × 10^−39^
SMA projection	Projection	FA_FWcorr_	−7.36 × 10^−3^ (5.60 × 10^−4^)	−13.15	5.75 × 10^−38^
M1 projection	Projection	FA_FWcorr_	−7.69 × 10^−3^ (6.04 × 10^−4^)	−12.72	1.01 × 10^−35^
PMd projection	Projection	FA_FWcorr_	−6.90 × 10^−3^ (5.43 × 10^−4^)	−12.71	1.01 × 10^−35^
***Main effects of APOE* ε4**					
ILF	Limbic	FW	6.70 × 10^−3^ (9.99 × 10^−4^)	6.71	1.03 × 10^−9^
Cingulum	Limbic	FW	5.36 × 10^−3^ (8.01 × 10^−4^)	6.70	1.03 × 10^−9^
Inferior temporal gyrus TC	Limbic	FW	5.58 × 10^−3^ (9.34 × 10^−4^)	5.97	7.47 × 10^−8^
Fornix	Limbic	FW	6.99 × 10^−3^ (1.22 × 10^−3^)	5.73	2.36 × 10^−7^
Superior occipital gyrus TC	Occipital TC	FW	6.32 × 10^−3^ (1.13 × 10^−3^)	5.61	3.99 × 10^−7^

Abbreviations: *APOE*, apolipoprotein E; FA_FWcorr_, free water–corrected fractional anisotropy; FW, free water; ILF, inferior longitudinal fasciculus; M1, primary motor cortex; PMd, dorsal premotor; PMv, ventral premotor; S1, primary somatosensory cortex; SE, standard error; SMA, supplementary motor area; TC, transcallosal.

### 3.3 *APOE* ε4 associations with FA_FWcorr_ and FW

Figure 2A illustrates that the effects of *APOE* ε4 on FA_FWcorr_ were limited (*n* = 8 tracts), with *APOE* ε4 carriers having higher FA_FWcorr_ longitudinally. The strongest association was observed in the temporoparietal superior longitudinal fasciculus association tract, as depicted in the spaghetti plot within Figure 2A (*P* = 4.82 × 10^−3^). FW was higher in *APOE* ε4 carriers in all tracts examined (Figure 2B) except the primary somatosensory cortex projection tract. As shown in Table 2, the strongest associations were observed for FW in limbic and occipital TC tracts. Statistics for all models can be found in Table S3. Cross‐sectional results were similar to those seen in longitudinal analyses. In cross‐sectional race‐stratified analyses, there was no association between *APOE* ε4 and FA_FWcorr_ or FW in non‐Hispanic Black adults. In non‐Hispanic White adults, there were limited differences in FA_FWcorr_, primarily in projection tracts, but more diffuse differences in FW metrics. Race‐stratified statistics can be found in Table S4.

### 3.4 Sex and *APOE* ε4 interactions

As summarized in Table 3, there were no statistically significant sex × *APOE* ε4 interactions on FA_FWcorr_ or FW when corrected for multiple comparisons in longitudinal analyses. In cross‐sectional race‐stratified analyses, there were no significant sex × *APOE* ε4 interactions for non‐Hispanic White or Black adults. Statistics for all models can be found in Tables S3 and S4.

**TABLE 3:** Top sex × *APOE* ε4 interactions on longitudinal white matter microstructure.

Tract	Tract type	Outcome	*β* (SE)	*z* value	*p* value
***Sex *×* APOE* ε4 *interactions***					
Superior occipital TC	Occipital TC	FW	−7.53 × 10^−3^ (2.27 × 10^−3^)	−3.31	0.053^a^
Cuneus TC	Occipital TC	FW	−7.21 × 10^−3^ (2.21 × 10^−3^)	−3.26	0.053^a^
Middle occipital TC	Occipital TC	FW	−7.68 × 10^−3^ (2.45 × 10^−3^)	−3.13	0.055^a^
Caudate to superior frontal	Projection	FA_FWcorr_	3.16 × 10^−3^ (1.10 × 10^−3^)	2.88	0.078^a^
Calcarine sulcus TC	Occipital TC	FW	−6.50 × 10^−3^ (2.26 × 10^−3^)	−2.88	0.078^a^

Abbreviations: *APOE*, apolipoprotein E; FA_FWcorr_, free water–corrected fractional anisotropy; FW, free water; SE, standard error; TC, transcallosal.

*Note*: Listed *P* values are corrected for multiple comparisons using the false discovery rate approach.

^a^ Uncorrected *P* value < 0.05.

### 3.5 Tract‐type associations

**FIGURE 3:**
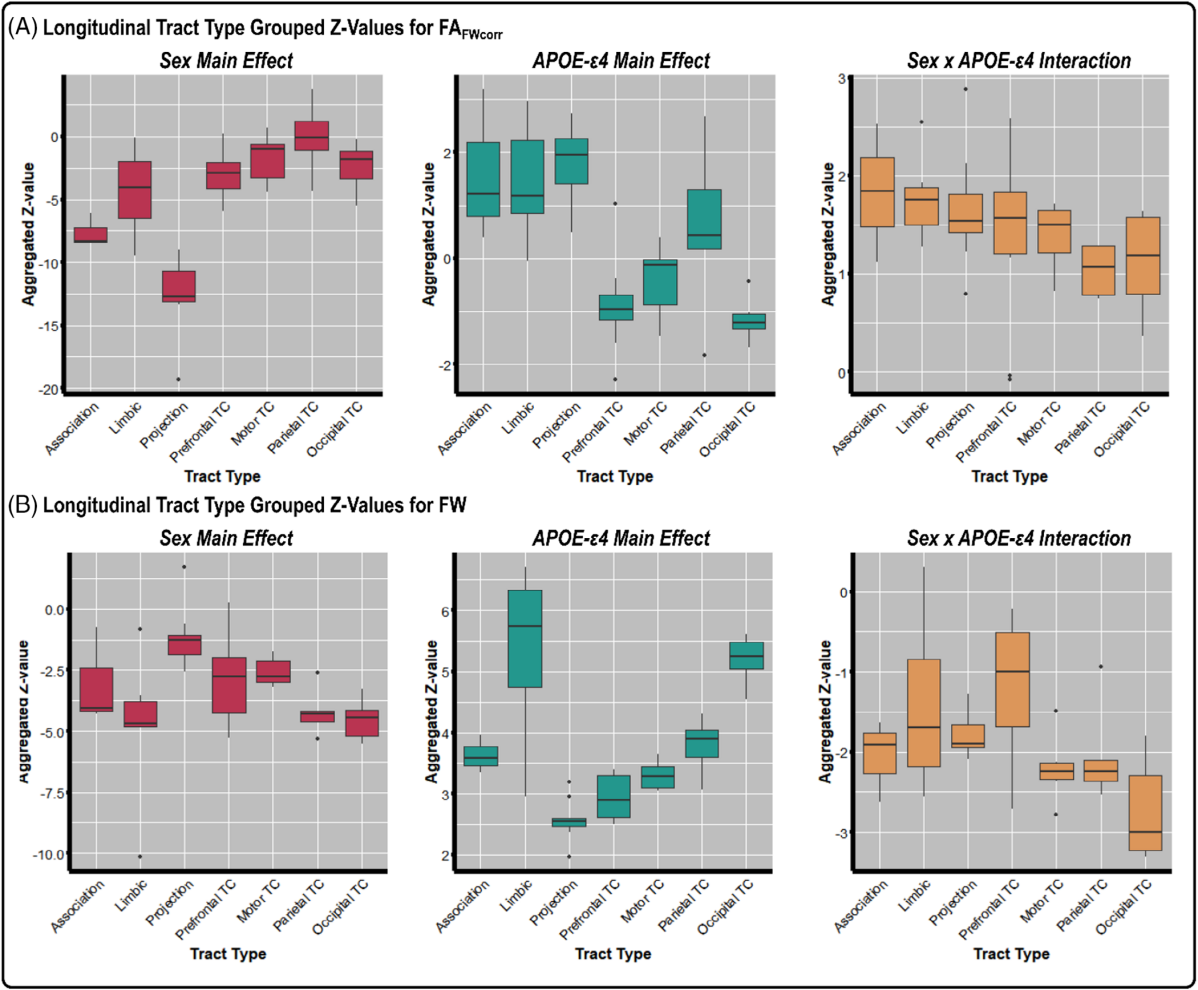
Aggregated tract‐type associations with changes in white matter microstructure. *z* values of individual tracts were grouped into the following tract types: association, limbic, projection, prefrontal TC, motor TC, parietal TC, and occipital TC for FA_FWcorr_ (A) and FW (B). The left, middle, and right columns display the sex main effect (red), *APOE* ε4 main effect (blue), and sex × *APOE* ε4 interaction (orange), respectively. *APOE*, apolipoprotein E; FA_FWcorr_, free water–corrected fractional anisotropy; FW, free water; TC, transcallosal.

Aggregating our longitudinal statistics for FA_FWcorr_ and FW across tract type (association, limbic, projection, prefrontal TC, motor TC, parietal TC, and occipital TC) may provide insight into basic brain changes that happen in the context of sex and *APOE* ε4 carrier status. Our aggregated longitudinal results for FA_FWcorr_ are shown in Figure 3A, in which we found significant tract‐type effects for sex (χ^2 ^= 29.93, *P* = 4.05 × 10^−5^) and *APOE* ε4 (*F* = 12.82, *P* = 4.23 × 10^−8^). For sex, we found that women exhibited lower FA_FWcorr_ in the projection tracts compared to the limbic, motor TC, occipital TC, parietal TC, and prefrontal TC tracts. For *APOE* ε4, we found that carriers had lower FA_FWcorr_ within the prefrontal TC, motor TC, parietal TC, and occipital TC tracts. There was no tract‐type significance for the sex × *APOE* ε4 interaction (*F* = 1.46, *P* = 0.22). Our results for FW are shown in Figure 3B, in which we found significant tract‐type effects for sex (χ^2 ^= 20.73, *P* = 2.05 × 10^−3^) and *APOE* ε4 (*F* = 35.68, *P* = 3.18 × 10^−6^). For sex, there was higher FW in females in the projection tracts compared to the occipital corpus callosum (CC) and parietal CC tracts. For *APOE* ε4 carriers, the limbic and occipital CC tracts had higher FW compared to the prefrontal CC and projection tracts.

### 3.6 Sensitivity analyses

Several sensitivity analyses can add further context to our sex and *APOE* ε4 findings on FA_FWcorr_ and FW. First, given that diagnosis has a convoluted role in sex, *APOE* ε4, and white matter microstructure, it is interesting to see the impact of adding a diagnosis covariate into our analyses. Results from this sensitivity analysis can be found in Table S3 and *P* values comparing these models can be found in Figure S1 in supporting information. While the results were almost identical for the sex main effect, there were some differences for *APOE* ε4. Further, comparing our FA_FWcorr_ and FW results to FA_CONV_ can tie our findings to prior literature in this space which used conventional DTI. Results from our FA_CONV_ analysis can be found in Table S3 and plots comparing the *P* values can be found in Figure S2 in supporting information. Our findings for sex and *APOE* ε4 were strongly associated for FA_FWcorr_ and FA_CONV_. Additional models were conducted to determine how conventional diffusivities (AxD_CONV_, RD_CONV_, MD_CONV_) and FW‐corrected diffusivities (AxD_FWcorr_, RD_FWcorr_, MD_FWcorr_) are impacted by sex and *APOE* ε4. All diffusivity statistics can be found in Table S5 in supporting information, which show the same general pattern as our FA_FWcorr_ and FW results.

## 4 DISCUSSION

This study leveraged nine well‐defined cohorts of older adults to characterize the effects of sex and *APOE* ε4 carrier status on the white matter microstructure of 48 tracts longitudinally using FW and FA_FWcorr_ metrics. We found that sex was most strongly associated with FA_FWcorr_, whereby females had lower FA_FWcorr_ over time than males. Sex differences were greatest in association and projection tracts. *APOE* ε4 status was globally associated with FW, most strongly in limbic and occipital TC tracts, such that *APOE* ε4 carriers had higher FW than non‐carriers over time. *APOE* ε4 status did not modify the relationships between sex on FW or FA_FWcorr_. This work adds to the field by exploring differences in white matter by sex and *APOE* ε4 carrier status on a larger scale than previously done.

This work advances the field by showing clear sex differences in white matter microstructure that were most pronounced in association and projection tracts. Sex differences were similar in both FA_conv_ and FA_FWcorr_. This was largely true of other diffusivity metrics (e.g., AxD_CONV_ and AxD_FWcorr_) as well, suggesting that observed microstructural changes were independent of extracellular water. Projection tracts connect the cortex to subcortical structures and help integrate sensory, motor, and cognitive functions. Similarly to previous work, we found that females had lower FA values in sensory and motor projections^10^ cross‐sectionally. Longitudinally, we found that while FA_CONV_ decreased with age, FA_FWcorr_ increased with age in both sexes and females showed a smaller change over time. We have previously found that age can be positively associated with FA_FWcorr_,^25^ which likely relates to FW contamination of FA_CONV_, making FA_FWcorr_ a more accurate measure of white matter microstructure. Previous work has consistently found that association tracts are affected in the aging process^10^, ^25^ and these tracts show a greater change in FA_CONV_ and FA_FWcorr_ in individuals with cognitive impairment relative to cognitively unimpaired adults.^48^ While some studies have shown that projection fibers are also associated with abnormal cognitive aging, these associations are less robust.^10^, ^25^, ^44^ Although higher FA is often suggested to be indicative of greater microstructural integrity, this metric is non‐specific and may not reflect greater integrity, particularly in areas with high volumes of crossing fibers. However, we also found that in tracts with sex differences in FW, females tended to have a smaller increase in FW than males longitudinally. Although FW has been proposed to have many possible neurobiological correlates, including atrophy, edema, and neuroinflammation, the etiology of FW is not agreed upon.^49^ We found that females in this study were less likely to be cognitively impaired at baseline and longitudinally than males, which could explain why they had lower FW values than males. Given that females are disproportionately affected by AD, our study suggests that white matter microstructural differences may contribute to this disparity. Future studies to determine whether observed changes in FW are compensatory and beneficial are necessary.

Despite the role of *APOE* in lipid homeostasis, previous studies have not consistently found an association between *APOE* ε4 and differences in white matter microstructure.^50^, ^51^, ^52^ In our study, *APOE* ε4 carrier status was associated with elevated FW, primarily in limbic and occipital TC tracts. There were fewer differences in FA_FWcorr_, and no differences in FA_CONV_. While there were global associations with other conventional diffusivity metrics (e.g., AxD_CONV_), these associations did not remain after FW correction. This suggests that observed associations in conventional metrics are driven by extracellular water rather than differences in the microstructural tissue itself. This is consistent with previous work in which we have shown that the FW measure, particularly in limbic tracts, is most sensitive to abnormal aging.^25^, ^44^ This study extends this work by showing that *APOE* ε4 carriers, who are known to be at greater risk for AD, show these same changes in the FW metric in limbic tracts. This suggests that processes that increase interstitial spaces, such as atrophy, inflammation, or edema, are present to a greater extent in *APOE* ε4 carriers in tracts known to be affected in AD. Given that sex did not moderate the association between *APOE* ε4 and FW, observed differences in *APOE* ε4 and AD risk by sex may not be driven by the same pathological processes that drive FW changes.

In this study, there were limited cross‐sectional associations between white matter microstructural metrics and either *APOE* ε4 or sex in non‐Hispanic Black adults, but more widespread associations in non‐Hispanic White adults. This study of FW and FA_FWcorr_ included the largest sample of non‐Hispanic Black adults that we are aware of. However, we were still underpowered to explore how *APOE* ε4 and sex affect white matter microstructure longitudinally, so race‐stratified results were only performed cross‐sectionally. It will be important to explore these associations longitudinally with larger samples in the future. We believe the observed racial differences in this study relate to sociocultural factors as opposed to biological differences between racial groups. Studies comparing racial and ethnic groups have suggested that social factors, including socioeconomic status^53^ and acculturation,^54^ affect white matter microstructure. Additional work to explore these pathways are warranted.

This study has several strengths. It used a large harmonized multisite diffusion MRI cohort that enhanced our statistical power to identify differences that would have been undetectable in smaller samples. We were able to look at 48 tractography templates that included association, limbic, projection, and TC tracts, allowing us to explore tract‐specific sex and *APOE* ε4 carrier differences in white matter microstructure. Additionally, we were able to account for partial volume effects by using FW and FW‐corrected diffusion metrics. This study also had several limitations. Although the use of single‐shell dMRI data allowed for the inclusion of more cohorts, there are inherent limitations that are overcome with multi‐shell data. Additionally, the neurodegenerative pattern of *APOE* ε2/ε4 carriers is complex. These carriers were considered *APOE* ε4 carriers in our analyses, but larger studies are needed to better determine how these individuals compare to other *APOE* ε4 carriers. While non‐Hispanic Black participants were included, they were primarily drawn from two cohorts and were less likely to be cognitively impaired at baseline and longitudinally than non‐Hispanic White participants, which is the opposite of what epidemiological studies show. Differences in cohort demographics may contribute to observed differences in white matter microstructure and could temper the conclusions that can be drawn. Additionally, although this study found notable differences in white matter microstructure, we are not able to determine the biological and social pathways that lead to these differences.

In this study, we demonstrate marked differences longitudinally in white matter microstructure by sex and *APOE* ε4 carrier status on a larger scale than previously done. Females and *APOE* ε4 carriers had differences in white matter microstructure compared to males and *APOE* ε4 non‐carriers. The markers of white matter microstructure and the tracts affected were not uniform, suggesting there may be different etiologies for the observed differences. Future studies that incorporate larger samples sizes of diverse participants, additional biomarkers, and information about social determinants of health are needed to clarify the reasons for the observed differences.

## CONFLICT OF INTEREST STATEMENT

S.C.J. has served on advisory boards for Enigma Biomedical and ALZPath in the past 2 years. A.J.S. receives support from multiple NIH grants (P30 AG010133, P30 AG072976, R01 AG019771, R01 AG057739, U19 AG024904, R01 LM013463, R01 AG068193, T32 AG071444, U01 AG068057, U01 AG072177, U19 AG074879, and U24 AG074855). He has also received support from Avid Radiopharmaceuticals, a subsidiary of Eli Lilly (in kind contribution of PET tracer precursor) and participated in scientific advisory boards (Bayer Oncology, Eisai, Novo Nordisk, and Siemens Medical Solutions USA, Inc) and an observational study monitoring board (MESA, NIH NHLBI), as well as external advisory committees for multiple NIA grants. He also serves as editor in chief of *Brain Imaging and Behavior*, a Springer‐Nature journal. B.A.L. serves as editor in chief of *SPIE Journal of Medical Imaging*. L.L.B. serves as deputy editor of *Alzheimer's & Dementia*. A.L.J. serves on the NINDS DIVERSE VCID and NINDS RECOVERY observational study monitoring boards. She serves on the scientific advisory committee for the Paul B. Beeson Emerging Leaders Career Development Program, American Federation for Aging Research. She serves on the Clin‐STAR Coordinating Center External Advisory Committee, the external advisory board for the Kansas Alzheimer's Disease Core Center, and the Alzheimer's Disease and Related Dementia Advisory Council. A.W.T. serves on the RHU‐SHIVA data safety monitoring board and the ADNI Steering Committee and Executive Committee and is a member of the World Dementia Council and the Alzheimer's Disease Initiative Global Technical Advisory Committee. T.J.H. serves on the scientific advisory board for Vivid Genomics. He serves as *Alzheimer's & Dementia: TRCI* deputy editor and the *Alzheimer's & Dementia* senior associate editor. J.E.W. received financial support from IONIS pharmaceuticals. A.P., A.S., D.Z., Y.Y., A.D., K.D.D., N.S., K.R.P., M.E.K., C.G., N.M.K., Z.L., T.Y., Y.K., L.D., K.A.G., F.E.C., S.L.R., L.L.B.H., Y.A., K.A., G.E., C.D., D.T., P.M.T., E.C.M., M.H., D.W., P.Z., K.S., M.A., W.K., S.A.B., J.S., D.A.B., S.M.R., D.B.A., ADNI, BIOCARD, and ADSP have no conflicts to disclose. The authors declare no conflicts of interest. Author disclosures are available in the supporting information.

## CONSENT STATEMENT

All participants provided informed consent in their respective cohort studies.

## Supporting information



alz14343‐sup‐0001‐Figures.docx

alz14343‐sup‐0002‐Tables.xlsx

alz14343‐sup‐0003‐ICMJE.pdf

## References

[1] Sachdev PS , Zhuang L , Braidy N , Wen W . Is Alzheimer's a disease of the white matter?. Curr Opin Psychiatry. 2013;26:244‐251. 23493128 10.1097/YCO.0b013e32835ed6e8

[2] Ihara M , Polvikoski TM , Hall R , et al. Quantification of myelin loss in frontal lobe white matter in vascular dementia, Alzheimer's disease, and dementia with lewy bodies. Acta Neuropathol. 2010;119:579‐589. 20091409 10.1007/s00401-009-0635-8 PMC2849937

[3] Simpson JE , Ince PG , Higham CE , et al. Microglial activation in white matter lesions and nonlesional white matter of ageing brains. Neuropathol Appl Neurobiol. 2007;33:670‐683. 17990995 10.1111/j.1365-2990.2007.00890.x

[4] Lee S , Viqar F , Zimmerman ME , et al. White matter hyperintensities are a core feature of Alzheimer's disease: evidence from the dominantly inherited Alzheimer network. Ann Neurol. 2016;79:929‐939. 27016429 10.1002/ana.24647 PMC4884146

[5] Bateman RJ , Xiong C , Benzinger TL , et al. Clinical and biomarker changes in dominantly inherited Alzheimer's disease. N Engl J Med. 2012;367:795‐804. 22784036 10.1056/NEJMoa1202753 PMC3474597

[6] Araque Caballero MA , Suarez‐Calvet M , Duering M , et al. White matter diffusion alterations precede symptom onset in autosomal dominant Alzheimer's disease. Brain. 2018;141:3065‐3080. 30239611 10.1093/brain/awy229 PMC6158739

[7] 2023 Alzheimer's disease facts and figures. Alzheimers Dement 2023;19:1598‐1695. 36918389 10.1002/alz.13016

[8] Nebel RA , Aggarwal NT , Barnes LL , et al. Understanding the impact of sex and gender in Alzheimer's disease: a call to action. Alzheimers Dement. 2018;14:1171‐1183. 29907423 10.1016/j.jalz.2018.04.008 PMC6400070

[9] Inano S , Takao H , Hayashi N , Abe O , Ohtomo K . Effects of age and gender on white matter integrity. AJNR Am J Neuroradiol. 2011;32:2103‐2109. 21998104 10.3174/ajnr.A2785 PMC7964377

[10] Cox SR , Ritchie SJ , Tucker‐Drob EM , et al. Ageing and brain white matter structure in 3,513 UK Biobank participants. Nat Commun. 2016;7:13629. 27976682 10.1038/ncomms13629 PMC5172385

[11] Isaac Tseng WY , Hsu YC , Chen CL , et al. Microstructural differences in white matter tracts across middle to late adulthood: a diffusion MRI study on 7167 UK Biobank participants. Neurobiol Aging. 2021;98:160‐172. 33290993 10.1016/j.neurobiolaging.2020.10.006

[12] O'Dwyer L , Lamberton F , Bokde AL , et al. Sexual dimorphism in healthy aging and mild cognitive impairment: a DTI study. PLoS One. 2012;7:e37021. 22768288 10.1371/journal.pone.0037021 PMC3388101

[13] Bergamino M , Keeling EG , Baxter LC , Sisco NJ , Walsh RR , Stokes AM . Sex differences in Alzheimer's disease revealed by free‐water diffusion tensor imaging and voxel‐based morphometry. J Alzheimers Dis. 2022;85:395‐414. 34842185 10.3233/JAD-210406 PMC9015709

[14] Chou KH , Cheng Y , Chen IY , Lin CP , Chu WC . Sex‐linked white matter microstructure of the social and analytic brain. Neuroimage. 2011;54:725‐733. 20633662 10.1016/j.neuroimage.2010.07.010

[15] Corder EH , Saunders AM , Strittmatter WJ , et al. Gene dose of apolipoprotein E type 4 allele and the risk of Alzheimer's disease in late onset families. Science. 1993;261:921‐923. 8346443 10.1126/science.8346443

[16] Neu SC , Pa J , Kukull W , et al. Apolipoprotein E genotype and sex risk factors for Alzheimer disease: a meta‐analysis. JAMA Neurol. 2017;74:1178‐1189. 28846757 10.1001/jamaneurol.2017.2188 PMC5759346

[17] Farrer LA , Cupples LA , Haines JL , et al. Effects of age, sex, and ethnicity on the association between apolipoprotein E genotype and Alzheimer disease. A meta‐analysis. APOE and Alzheimer Disease Meta Analysis Consortium. JAMA. 1997;278:1349‐1356. 9343467

[18] Bartzokis G , Lu PH , Geschwind DH , et al. Apolipoprotein E affects both myelin breakdown and cognition: implications for age‐related trajectories of decline into dementia. Biol Psychiatry. 2007;62:1380‐1387. 17659264 10.1016/j.biopsych.2007.03.024

[19] Reinvang I , Espeseth T , Westlye LT . APOE‐related biomarker profiles in non‐pathological aging and early phases of Alzheimer's disease. Neurosci Biobehav Rev. 2013;37:1322‐1335. 23701948 10.1016/j.neubiorev.2013.05.006

[20] Goltermann J , Repple J , Redlich R , et al. Apolipoprotein E homozygous epsilon4 allele status: effects on cortical structure and white matter integrity in a young to mid‐age sample. Eur Neuropsychopharmacol. 2021;46:93‐104. 33648793 10.1016/j.euroneuro.2021.02.006

[21] Glymour MM , Manly JJ . Lifecourse social conditions and racial and ethnic patterns of cognitive aging. Neuropsychol Rev. 2008;18:223‐254. 18815889 10.1007/s11065-008-9064-z

[22] Wilkins CH , Windon CC , Dilworth‐Anderson P , et al. Racial and ethnic differences in amyloid PET positivity in individuals with mild cognitive impairment or dementia: a secondary analysis of the Imaging Dementia‐Evidence for Amyloid Scanning (IDEAS) cohort study. JAMA Neurol. 2022;79:1139‐1147. 36190710 10.1001/jamaneurol.2022.3157 PMC9531087

[23] Deters KD , Napolioni V , Sperling RA , et al. Amyloid PET imaging in self‐identified non‐Hispanic Black participants of the anti‐amyloid in asymptomatic Alzheimer's disease (A4) study. Neurology. 2021;96:e1491‐e500. 33568538 10.1212/WNL.0000000000011599 PMC8032379

[24] Pasternak O , Sochen N , Gur Y , Intrator N , Assaf Y . Free water elimination and mapping from diffusion MRI. Magn Reson Med. 2009;62:717‐730. 19623619 10.1002/mrm.22055

[25] Archer DB , Schilling K , Shashikumar N , et al. Leveraging longitudinal diffusion MRI data to quantify differences in white matter microstructural decline in normal and abnormal aging. Alzheimers Dement. 2023;15:e12468. 10.1002/dad2.12468 PMC10540270 37780863

[26] Bergamino M , Walsh RR , Stokes AM . Free‐water diffusion tensor imaging improves the accuracy and sensitivity of white matter analysis in Alzheimer's disease. Sci Rep. 2021;11:6990. 33772083 10.1038/s41598-021-86505-7 PMC7998032

[27] Chad JA , Pasternak O , Salat DH , Chen JJ . Re‐examining age‐related differences in white matter microstructure with free‐water corrected diffusion tensor imaging. Neurobiol Aging. 2018;71:161‐170. 30145396 10.1016/j.neurobiolaging.2018.07.018 PMC6179151

[28] Sexton CE , Kalu UG , Filippini N , Mackay CE , Ebmeier KP . A meta‐analysis of diffusion tensor imaging in mild cognitive impairment and Alzheimer's disease. Neurobiol Aging. 2011;32:e5‐18. 2322. 10.1016/j.neurobiolaging.2010.05.019 20619504

[29] Stricker NH , Schweinsburg BC , Delano‐Wood L , et al. Decreased white matter integrity in late‐myelinating fiber pathways in Alzheimer's disease supports retrogenesis. Neuroimage. 2009;45:10‐16. 19100839 10.1016/j.neuroimage.2008.11.027 PMC2782417

[30] Nir TM , Jahanshad N , Villalon‐Reina JE , et al. Effectiveness of regional DTI measures in distinguishing Alzheimer's disease, MCI, and normal aging. Neuroimage Clin. 2013;3:180‐195. 24179862 10.1016/j.nicl.2013.07.006 PMC3792746

[31] jr Jack CR, , Bernstein MA , Fox NC , et al. The Alzheimer's Disease Neuroimaging Initiative (ADNI): MRI methods. J Magn Reson Imaging. 2008;27:685‐691. 18302232 10.1002/jmri.21049 PMC2544629

[32] Ferrucci L . The Baltimore Longitudinal Study of Aging (BLSA): a 50‐year‐long journey and plans for the future. J Gerontol: Series A. 2008;63:1416‐1419. 10.1093/gerona/63.12.1416 PMC5004590 19126858

[33] Albert M , Soldan A , Gottesman R , et al. Cognitive changes preceding clinical symptom onset of mild cognitive impairment and relationship to ApoE genotype. Curr Alzheimer Res. 2014;11:773‐784. 25212916 10.2174/156720501108140910121920 PMC4163954

[34] Besser L , Kukull W , Knopman DS , et al. Version 3 of the National Alzheimer's Coordinating Center's Uniform Data Set. Alzheimer Dis Assoc Disord. 2018;32:351‐358. 30376508 10.1097/WAD.0000000000000279 PMC6249084

[35] Bennett DA , Buchman AS , Boyle PA , Barnes LL , Wilson RS , Schneider JA . Religious orders study and rush memory and aging project. J Alzheimers Dis. 2018;64:S161‐S89. 29865057 10.3233/JAD-179939 PMC6380522

[36] Barnes LL , Shah RC , Aggarwal NT , Bennett DA , Schneider JA . The Minority Aging Research Study: ongoing efforts to obtain brain donation in African Americans without dementia. Curr Alzheimer Res. 2012;9:734‐745. 22471868 10.2174/156720512801322627 PMC3409294

[37] Makkinejad N , Evia AM , Tamhane AA , et al. ARTS: a novel in‐vivo classifier of arteriolosclerosis for the older adult brain. Neuroimage Clin. 2021;31:102768. 34330087 10.1016/j.nicl.2021.102768 PMC8329541

[38] Jefferson AL , Gifford KA , Acosta LM , et al. The Vanderbilt Memory & Aging Project: study design and baseline cohort overview. J Alzheimers Dis. 2016;52:539‐559. 26967211 10.3233/JAD-150914 PMC4866875

[39] Johnson SC , Koscik RL , Jonaitis EM , et al. The Wisconsin Registry for Alzheimer's Prevention: a review of findings and current directions. Alzheimers Dement. 2017;10:130‐142. 10.1016/j.dadm.2017.11.007 PMC5755749 29322089

[40] Cai LY , Yang Q , Hansen CB , et al. PreQual: an automated pipeline for integrated preprocessing and quality assurance of diffusion weighted MRI images. Magn Reson Med. 2021;86:456‐470. 33533094 10.1002/mrm.28678 PMC8387107

[41] Schilling KG , Blaber J , Huo Y , et al. Synthesized b0 for diffusion distortion correction (Synb0‐DisCo). Magn Reson Imaging. 2019;64:62‐70. 31075422 10.1016/j.mri.2019.05.008 PMC6834894

[42] Avants BB , Epstein CL , Grossman M , Gee JC . Symmetric diffeomorphic image registration with cross‐correlation: evaluating automated labeling of elderly and neurodegenerative brain. Med Image Anal. 2008;12:26‐41. 17659998 10.1016/j.media.2007.06.004 PMC2276735

[43] Archer DB , Moore EE , Shashikumar N , et al. Free‐water metrics in medial temporal lobe white matter tract projections relate to longitudinal cognitive decline. Neurobiol Aging. 2020;94:15‐23. 32502831 10.1016/j.neurobiolaging.2020.05.001 PMC7483422

[44] Yang Y , Schilling K , Shashikumar N , et al. White matter microstructural metrics are sensitively associated with clinical staging in Alzheimer's disease. Alzheimers Dement. 2023;15:e12425. 10.1002/dad2.12425 PMC10192723 37213219

[45] Yang Y , Sathe A , Schilling K , et al. A deep neural network estimation of brain age is sensitive to cognitive impairment and decline. Pac Symp Biocomput. 2024;29:148‐162. 38160276 PMC10764074

[46] Archer DB , Moore EE , Pamidimukkala U , et al. The relationship between white matter microstructure and self‐perceived cognitive decline. Neuroimage Clin. 2021;32:102794. 34479171 10.1016/j.nicl.2021.102794 PMC8414539

[47] Beer JC , Tustison NJ , Cook PA , et al. Longitudinal ComBat: a method for harmonizing longitudinal multi‐scanner imaging data. Neuroimage. 2020;220:117129. 32640273 10.1016/j.neuroimage.2020.117129 PMC7605103

[48] Shafer AT , Williams OA , Perez E , et al. Accelerated decline in white matter microstructure in subsequently impaired older adults and its relationship with cognitive decline. Brain Commun. 2022;4:fcac051. 35356033 10.1093/braincomms/fcac051 PMC8963308

[49] Maillard P , Fletcher E , Singh B , et al. Cerebral white matter free water: a sensitive biomarker of cognition and function. Neurology. 2019;92:e2221‐e2231. 30952798 10.1212/WNL.0000000000007449 PMC6537135

[50] Srisaikaew P , Chad JA , Mahakkanukrauh P , Anderson ND , Chen JJ . Effect of sex on the APOE4‐aging interaction in the white matter microstructure of cognitively normal older adults using diffusion‐tensor MRI with orthogonal‐tensor decomposition (DT‐DOME). Front Neurosci. 2023;17:1049609. 36908785 10.3389/fnins.2023.1049609 PMC9992882

[51] Adluru N , Destiche DJ , Lu SY , et al. White matter microstructure in late middle‐age: effects of apolipoprotein E4 and parental family history of Alzheimer's disease. Neuroimage Clin. 2014;4:730‐742. 24936424 10.1016/j.nicl.2014.04.008 PMC4053649

[52] Wang R , Fratiglioni L , Laukka EJ , et al. Effects of vascular risk factors and APOE ε4 on white matter integrity and cognitive decline. Neurology. 2015;84:1128‐1135. 25672924 10.1212/WNL.0000000000001379 PMC4371409

[53] Hall JR , Johnson LA , Zhang F , et al. Using fractional anisotropy imaging to detect mild cognitive impairment and Alzheimer's disease among Mexican Americans and non‐Hispanic Whites: a HABLE Study. Dement Geriatr Cogn Disord. 2021;50:266‐273. 34569492 10.1159/000518102 PMC8559764

[54] Fleischman DA , Arfanakis K , Zhang S , et al. Acculturation in context and brain health in older Latino adults: a diffusion tensor imaging study. J Alzheimers Dis. 2023;95:1585‐1595. 37718813 10.3233/JAD-230491 PMC10599486

