# Supplementary material for: Sex and APOE ε4 allele differences in longitudinal white matter microstructure in multiple cohorts of aging and Alzheimer's disease
Source: Alzheimers Dement. 2024 Dec 22;21(1):e14343. doi: 10.1002/alz.14343 (PMC11781133; doi:10.1002/alz.14343)
Supplement: Supplementary file 1 — alz14343‐sup‐0001‐Figures.docx [file ALZ-21-e14343-s003.docx]

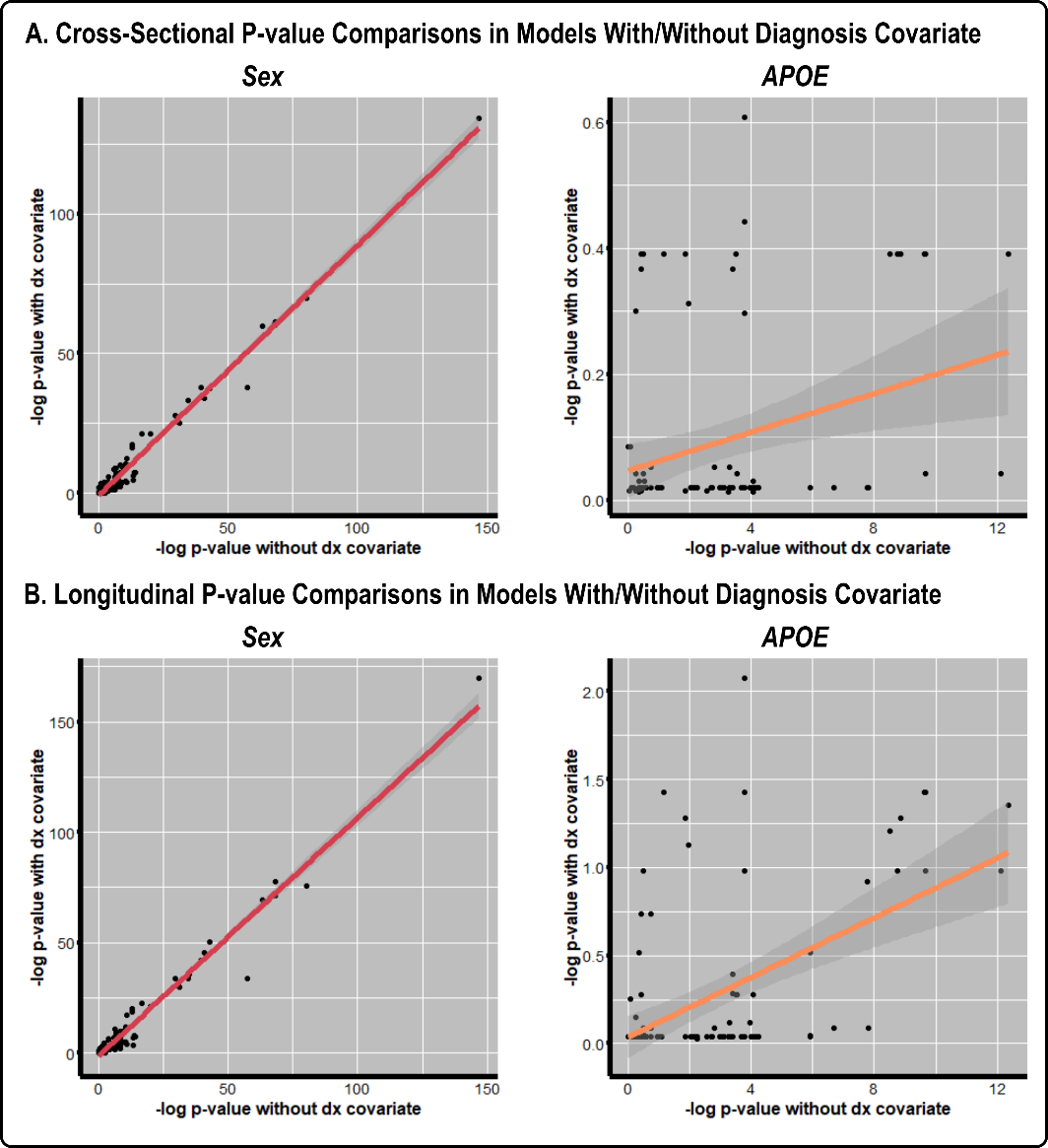


**Supplemental Figure 1.** Cross-sectional and longitudinal results with and without the inclusion of a diagnosis covariate.


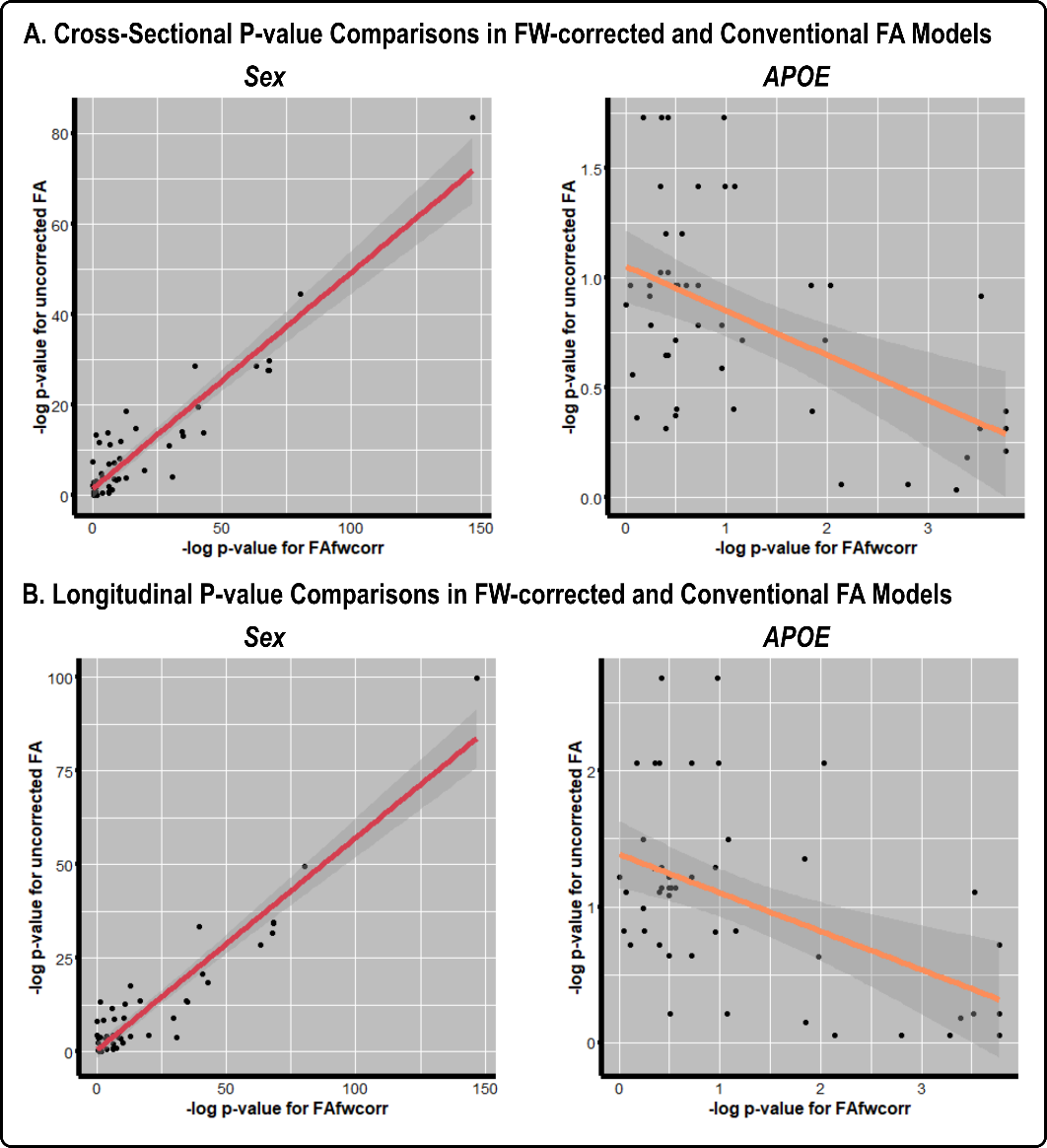


**Supplemental Figure 2.** Comparison of p-values obtained when using FA_FWcorr_ compared to uncorrected FA (i.e., FA_CONV_).
